# Cognitive control, bedtime patterns, and testing time in female adolescent students: behavioral and neuro-electrophysiological correlates

**DOI:** 10.3389/fpubh.2023.1022731

**Published:** 2023-06-19

**Authors:** Amedeo D’Angiulli, Gabriel Byczynski, Wei-Hsien Yeh, George Garrett, Gary Goldfield, Peter Devenyi, Tibor Devenyi, Gerry Leisman

**Affiliations:** ^1^Neuroscience of Imagination, Cognition and Emotion Research Lab, Department of Neuroscience, Carleton University, Ottawa, ON, Canada; ^2^School of Psychology, Institute of Neuroscience, Trinity College Dublin, Dublin, Ireland; ^3^Centre for Study and Treatment of Circadian Rhythms, Douglas Mental Health University Institute, McGill University, Montreal, QC, Canada; ^4^Ministry of Children and Family Development, Victoria, BC, Canada; ^5^Children’s Hospital of Eastern Ontario Research Institute, Ottawa, ON, Canada; ^6^Movement and Cognition Laboratory, Department of Physical Therapy, Faculty of Social Welfare and Health Science, University of Haifa, Mount Carmel, Israel; ^7^Department of Neurology, Institute for Neurology and Neurosurgery, University of the Medical Science, Havana, Cuba

**Keywords:** circadian rhythm, electroencephalography, sleep patterns, adolescent sleep, social jet lag, school start time, bed time behaviors

## Abstract

**Introduction:**

Shorter and/or disrupted sleep during adolescence is associated with cognitive and mental health risks, particularly in females. We explored the relationship between bedtime behavior patterns co-varying with Social Jet Lag (SJL) and School Start Times (SST) and neurocognitive performance in adolescent female students.

**Methods:**

To investigate whether time of day (morning vs. afternoon), early SSTs and days of the school week can be correlated with neurocognitive correlates of sleep insufficiency, we recruited 24 female students aged 16–18 to report sleep logs, and undergo event-related electroencephalographic recordings on Monday, Wednesday, mornings, and afternoons. Using a Stroop task paradigm, we analyzed correlations between reaction times (RTs), accuracy, time of day, day of week, electroencephalographic data, and sleep log data to understand what relationships may exist.

**Results:**

Participants reported a 2-h sleep phase delay and SJL. Stroop interference influenced accuracy on Monday and Wednesday similarly, with better performance in the afternoon. For RTs, the afternoon advantage was much larger on Monday than Wednesday. Midline Event-Related Potentials (ERPs) yielded higher amplitudes and shorter latencies on Wednesday morning and Monday afternoon, in time windows related to attention or response execution. A notable exception were delayed ERP latencies on Wednesday afternoon. The latter could be explained by the fact that delta EEG waves tended to be the most prominent, suggesting heightened error monitoring due to accumulating mental fatigue.

**Discussion:**

These findings provide insights into the interaction between SJL and SST and suggest evidence-based criteria for planning when female adolescents should engage in cognitive-heavy school activities such as tests or exams.

## 1. Introduction

### 1.1. Background

As children transition into adolescence, they typically prefer later sleeping and waking up times (1); this preference conflicts with school start times (SSTs), which usually begins in the early morning (2, 3). Conflict with early SST increases the risk of negative health and achievement outcomes, such as poor performance at school, daytime sleepiness, increased moodiness, and increased risk of substance use, including the use of stimulants and alcohol (4–8). A substantial body of research shows that the risks are much higher in adolescent and young adult female students (9). A particular sex-specific concern is that females typically need and sleep more than males, and sleep deprivation in general shows dramatic consequences for several neurocognitive outcomes and performance (10–12).

A body of literature suggests that sleep and wake preferences are influenced by circadian rhythms, which in turn influence cognitive performance (13–15). In particular, peak or optimal performance periods for cognitive tasks, including inhibitory thought and action, correlate with levels of circadian arousal (14), and younger adults, having mainly a neutral or evening preference, tend to perform significantly better on cognitive tasks during the afternoon compared to older adults who have a morning-type circadian preference.

Social factors interact significantly with sleep timing, this relationship is often framed in terms of sleep behavior differences between working days and free days (16). Namely, the sleep–wake cycle is also significantly influenced by lifestyle effects, working, or studying hours, and personal time, and not strictly by physiological regulation (17). Lifestyle schedules which conflict with natural circadian rhythms may result in a chronic disruption leading to misaligned circadian phase, otherwise known as social jetlag (SJL) (18). That is, SJL is a proxy which is assumed to reflect the psychophysical correlate of social and biological misalignment. One event which may be related to the effects of SJL is the change in sleep duration from Sunday to Monday in individuals who typically have a Monday–Friday work week. Since sleep duration from Sunday to Monday is greatly reduced due to an earlier wake-up time on Monday, and later bedtime on Sunday, a cycle of sleep deprivation and chronological misalignment might be iteratively reinforced across the sequence of week-weekend periods (19). Indeed, the effect of SST could be conceived as an influencing exogenous factor (i.e., not determined by individual’s endogenous factors such as voluntary behaviors and preferences) on chronobiological phase, which may indeed be enforcing a perpetuating SJL. Critically, SJL may be more detrimental to academic performance in adolescent female students, since their shift to evening-ness occurs earlier than in adolescent males because of earlier pubertal development (20).

Surprisingly, not much research has focused on changes in sleep patterns between Monday and Friday. It is still unclear whether sleep loss increases cumulatively during school weekdays. The latter hypothesis is usually linked with the classic tenet of the so-called *sleep debt* in sleep–wake regulating models (21). During daytime wakefulness, sleep pressure grows exponentially reaching its peak in the late evening when, after the transition to sleep, it starts an exponential decay. If wakefulness is voluntarily extended beyond the night switching point or threshold, an additional buildup of sleep pressure occurs. This additional buildup is traditionally theorized as a process of accumulation of “sleep debt” that is “paid” back during the following longer and more intense recovery sleep. However, simulation modeling studies of sleep times on weekdays and weekends do not find evidence for the accumulation of this sleep debt (22).

Particularly, Putilov and coworkers (12) tested whether week-day accumulation of sleep debt in student populations could be linked specifically to insufficiency and suboptimal levels of sleep through the specific effect of early SSTs, independent of other SJL effects. Their results, however, showed that weekend sleep after late SST was not shorter than that after early SST. A further study suggested that during weekdays there seems to be simply irrecoverable loss of some amount of night sleep due to early wakeups in those days (23).

Thus, while some evidence supports the hypothesis that circadian preferences (chronotypes) are associated with cognitive performance, especially in adolescents, other lines of evidence highlight that sleep patterns of adolescents vary adaptively throughout the week, predominantly through behavior associated with homeostatic regulation (24), and especially during the weekends when youth tend to stay up later (2, 16). Accordingly, it is possible that changes in bedtime behavior, rather than chronotypes, could be more of an influencing factor on the differences on cognitive performance during early morning, especially at the beginning of the week, i.e., on Monday, as compared to later in the work/school week (1).

Current understanding of adolescent student’s wake/sleep cycle and how it relates to neurocognitive functions and related academic performance is still limited by the scarcity of objective data regarding their bedtime behavioral patterns during the school weekdays. Typically, research has focused on the contrast between self-reports or questionnaires on weekdays versus weekends, assuming both time intervals as homogeneous undifferentiated blocks. In other words, a default assumption has been that SJL remains approximately the same throughout the school week. However, there is little evidence to support such an assumption. Although some research has examined the behavioral component of testing (i.e., situational context, specificity, etc.) and the effect of Monday and early week as opposed to other weekdays (in other contexts known as the “Monday effect”) (25), limited research has been conducted to examine possible underlying neurocognitive correlates of day of testing during school week. In particular, very little direct objective evidence links the covariation of day of testing with SJL and SSTs, and with *in-vivo* brain functioning in adolescents in school context. Gaining more knowledge and evidence on these relationships could have vital implications for the multiple allied fields within the learning sciences, since there seems to be no scientific guidelines available to teachers and educators to plan when would be the best time for adolescents to be engaged in cognitive-heavy school activities such as tests and exams.

### 1.2. Present study

Review of most recent neurocognitive studies reveals mixed, and nuanced, patterns of findings which do not neatly fit a single or simple explanation. Some studies suggested that sleep deprivation seems particularly to impair complex executive functions involving the prefrontal regions (26–28). Specifically, SJL seems to be associated with speed of processing in adults (29). However, some other studies failed to demonstrate such effects (30–32). Unlike subjective poor sleep quality (33, 34) or sleep loss (35), neither chronotype nor social jetlag were found to be associated with decision-making (29). Nevertheless, evidence suggests that chronotype may have a greater influence on performance at non-optimal times of the day (36).

A possible account for the mixed neurocognitive findings (37) is that there might be two different, main types of concurrent interacting mechanisms with different contributions to sleep loss. Some sleep loss may be mainly associated with SJL (38, 39) and may impair specifically aspects of cognitive control related to speed of processing and response (arousal, vigilance, attention, and inhibition). At the same time, another portion of sleep loss may be insufficiency due to behavioral homeostatic regulation which would impair more complex cognitive control processes linked with performance accuracy, including aspects of executive control (i.e., conflict sensitivity, error monitoring); the latter might be associated with suboptimal quantity and quality of sleep induced by early SSTs (40). An undetermined aspect of this explanation is which shape the SJL-SST interaction would take, hence, how it would reflect in the cognitive performance outcomes; would their influences on the observed performance interact additively? Else, would they interact synergistically, antagonistically, or even show a combination of different interactive relationships depending on the period of the week?

With the purpose of filling some gaps in literature, we explored the relationship between bedtime behavior patterns co-varying with SJL and SSTs (as reflected in daily and weekly times of testing) and neurocognitive performance in adolescent female students.

To estimate by proxy effects of delayed sleep pattern and circadian arousal associated with both SJL and SSTs, we collected adolescent’s weekly bedtime behavior logs. Chronobiology evidence suggests that, from Sunday to Monday, there is an instantaneous and significant shift of the sleep phase to an earlier time of day, similar to what happens when flying through 2 time zones in an easterly direction (41). Therefore, it is reasonable to expect that at the beginning of the week, adolescent students with a late chronotype should have the highest SJL on Monday morning. At the same time, sleep deprivation may be weakly manifested because adolescents may tend to sleep off on weekends (42). In contrast, the greatest sleep deprivation theoretically accumulates toward the end of the school week (43). That is, over the course of the week, an effect of accumulating sleep deprivation should be detectable by mid-week (i.e., Wednesday). Therefore, we hypothesized that SJL should be highest on Monday, and it should decline by Wednesday (i.e., SJL-Wed < SJL-Mo), but concurrently, *SST-related sleep deprivation* (henceforth SST-SD) would increase from Monday to Wednesday or remain at similar sustained levels in both days.

To examine whether SJL and SST-SD are indeed associated with different neurocognitive outcomes respectively, we assessed adolescents’ performance [accuracy and reaction time (RT)] in the morning and afternoon of Monday and Wednesday during ordinary school weeks/days, by using a specific variant of the Stroop task –the color-word judgment task – which was appropriate for probing *cognitive control*. Therefore, this Stroop variant provides a reasonably good model for cognitive performance during complex daily school activities involving higher cognitive processes such as, for example, taking multiple-choice tests or exams.

Within the cognitive field, there is consensus in interpreting relatively higher behavioral accuracy rates and faster RTs as best/optimal cognitive performance (44–46). Conversely, Stroop interference is defined as the extent to which the Stroop manipulation reduces performance. For adolescents, afternoons are more likely to align with the optimal time of late chronotype but are relatively ineffectual for early chronotype (47, 48). Therefore, we expected that Stroop interference on RTs and accuracy in our sample should always be higher in the Morning (AM) than in the Afternoon (PM). In addition, on the hypothesis that SJL would exert the main influence on behavioral performance, Stroop interference on both RTs and accuracy should be higher on Monday than on Wednesday.

Our critical test was whether, and if so, how SJL may interact with SST-SD. If SJL and SST-SD interacted non-additively, then there should be differences between Monday and Wednesday in the AM-*vs*-PM effect sizes. In addition, if SJL influence were predominantly associated with processing speed, while SST-SD influence were predominantly associated with accuracy, the differences between Monday and Wednesday in AM-*vs*-PM effect sizes should go in opposite directions in relation to RTs and accuracy, respectively, because they would mirror their respective time-trends. Specifically, we should observe that, for RTs, the AM-*vs*-PM difference in Stroop interference effect size should be larger on Monday than Wednesday [i.e., Monday (AM-*vs*-PM) > Wednesday (AM-*vs*-PM)] because SJL is higher on Monday declining progressively through Wednesday. However, the opposite pattern should occur for accuracy [i.e., Wednesday (AM-*vs*-PM) > Monday (AM-*vs*-PM)], since from Monday to Wednesday SST-SD would increase or remain at a sustained plateau level.

The latter “probing hypotheses” would allow us to gain novel insights into the processes in question no matter what the actual pattern of results is. Indeed, alternative possible outcomes might include independent additive influences, so that the effect size of the AM-*vs*-PM differences are consistently similar across Monday and Wednesday for both RTs and accuracy; alternatively, results could yield a more complex hybrid pattern showing a combination of different non-additive or/and additive interactions, which could differ for RTs and accuracy, respectively.

In addition to cognitive performance, we measured concurrent event-related potentials (ERPs) in the school setting. Given their superior temporal resolution, ERPs offer the opportunity to measure with relative precision the neural correlates underlying the behavioral Stroop performance over time. There is substantial consensus that functional neuroimaging correlates (i.e., as measured by fMRI and EEG/ERPs) of this particular or similar versions of the Stroop reliably and consistently point to a midline frontoparietal network associated with cognitive control [see comprehensive review in (49)].

In the present study, Stroop-concurrent ERPs from midline frontal (Fz), Central (Cz) and Parietal (Pz) electrodes were examined in correspondence with three well-known waveform time windows which have featured prominently in the literature on cognitive control: the P300 (250–400 ms), the N450 (400–700 ms), and the Sustained Potentials or SP (700–1,000 ms). The literature shows converging evidence that these ERP signatures generally yield higher amplitudes when associated with efficient, optimal performance, but in contrast delayed latencies when associated with performance deficits (50–52). Furthermore, the combination of higher amplitude plus delayed latency is generally interpreted in terms of increased cognitive load (53, 54) and when this overlaps or follows task response it can be considered as a marker of “neural effort” (55).

In keeping with the chronobiological rationale, we hypothesized that the patterns of results for the ERPs should be consistent with the pattern of results for the cognitive performance. Specifically, we expected ERPs to have longer (i.e., slower) latencies and lower amplitudes in the morning than in the afternoon and on Monday as compared to Wednesday.

Finally, we included an explorative analysis of midline frontal event-related EEG band-power, which might clarify the specific processes underlying the adolescents’ differences in ERPs and cognitive performance.

## 2. Materials and methods

### 2.1. Participants

Because of possible confounds due to sex, racial/ethnic and socioeconomic disparities in adolescents’ sleep duration (56) we sampled a homogeneous group of students. We initially recruited and included 28 participants, however due to incomplete (e.g., sleep logs and/or behavioral) or low-quality signal (i.e., high EEG noise), we discarded data from four participants. The final sample included 24 grade 11 and 12 Caucasian female high school students, ranging from ages 16 to 18 (Mean age = 16.9, SD = 0.4), were recruited from a semirural middle-sized town (population ~ 65,000) in Southwestern Canada. The determination of the initial sample size for a within-subject matched pairs design was based on our own and others’ previous experimental neurocognitive research using Stroop ERPs which usually in lab conditions obtained high effect sizes (i.e., d > 0.80). With 28 participants we predicted a minimum power of 83% (with two-tailed significance criterion of 0.05). As a result of the reduction to n = 24, although the minimum expected power declined to 77%, it was still at a quite good level [see Table 2.3.5, page 36, in (57)]. Participants were of European descent, living in the same neighborhood, and coming from a middle-high socioeconomic family background corresponding to class I-II on the Hollingshead’s socioeconomic inventory (58). Students were approached in the context of an orientation seminar outreach for targeted high schools and received course credits for their participation in the context of social studies curriculum. For four of the participants, we could only use self-report or/and behavioral data (see below). Signed consent for participation was obtained from the parents and the students. This study was approved by the ethics committees of British Columbia’s School District 73, Thompson Rivers University and Carleton University in accordance with the 1964 Declaration of Helsinki ethical standards and the Tri-Council Policy Statement.^1^

### 2.2. Sleep logs

Participants kept a sleep log for two consecutive weeks preceding neurocognitive testing, between the school March break and the easter week, with no holidays in between. Adolescent self-reports of sleep have a high correspondence with polysomnographic measures of sleep (kappa = 0.87), with high sensitivity (92.3%) and specificity (95.6%) (59), and can be more reliable than parent-reports (60). The sleep log included questions selected from a sleep clinic screening questionnaire battery validated in the general population from which the present sample was drawn (61); selection was in part determined by response compliance, all selected questions yielded compliance rates between 79 and 100% (19/24 and 24/24) responses on each item in the 2 weeks prior testing. The questions used were: (1) “What time did you turn off the light to go to sleep?” which defined the self-report measure of *fell asleep time proxy*; (2) “What time did you get up?” which defined the self-report measure of *wake-up time*; and (3) “Number of hours of actual sleep?” which defined the self-report measure of *hours of sleep per night (sleep hours)*. The sleep log included the following control questions: (a) How much coffee or drinks with caffeine (for example, Coca-Cola, Mountain Dew, etc.) today? How much after 4 pm? (b) How much did you smoke today? How much within 2 h of bedtime? (c) How much alcohol today? How much within 2 h of bedtime? (d) Did you exercise today? What time? For how long?

In addition, participants could use a checkbox at the end of the log forms to indicate whether anything unusual was noticed in their bodily functions and their state of health (participants were verbally instructed that next to the checkbox they could, if willing, volunteer information about their menstrual cycle). Responses to these questions were very sparse, only from 8 to 15% of the participants and, consequently, unbalanced (unequally distributed) across the group testing conditions to which the participants were assigned (see below). We attempted to run nonparametric association analyses testing for associations to somehow cluster the variables in composite rankings and then use these as control covariates in the main analyses involving sleep logs, behavioral and ERP data. However, all preliminary analyses that we attempted were unsuccessful and showed little variability or unreliable intercorrelations in caffeine, nicotine, alcohol, napping, or exercise or reports of unusual health occurrences. Therefore, given the poor quality and reliability of this data, they were not further considered (that is, they were not entered in the main analyses making up the bulk of the reporting in this paper). This is a limitation of our data and analysis which was however partly addressed by our sampling approach aimed at insuring participants’ comparability, see *Stroop testing procedure and context* section.

Three further questions, which were used to cross-check validity of the log reported data, were asked during the 2 weeks of testing. These additional questions prompted the participants to provide an estimate of the approximate times when participants thought they were “winding down,” “falling asleep” and “felt fully awake.”

During the 2 weeks of neurocognitive testing, we also asked students to rate on a 5-point Likert scale the following experiences: (1) Quality of sleep during night; (2) Alertness during the day; compliance rates were 75 and 79%, respectively.

Although we did not explicitly and directly instruct to use alarms or clocks to fill out the logs, all participants reported they did (mostly on their cell phones), either on their own or after being awoken by a parent.

### 2.3. Stroop testing and context

The design, sampling and testing schedule of the present study is detailed in Supplementary Table 1. Participants were randomly assigned into two groups of 12 and were designated as either the “morning” test group (9:30 am–10:30 am) or the “afternoon” test group (12:15 pm–2:15 pm) using a parallel group mixed design. Thus, the day of the week was the within-subjects factor, while testing time was a between-subjects factor. Extensive pilot studies showed that the best way to retain the same participants in the two scheduled sessions at test and retest (ensuring low attrition at retest) for these students, was to schedule the testing sessions in the same days in which participants attended courses within a particular grade (in this case, a pre-university credit high school psychology course which was offered twice a week). Therefore, these logistics constrained our testing scheduling and, consequently, our experimental design. Within each group, participants were assigned to one of two subgroups depending on their school grade; identical versions of the Stroop tests were administered to both subgroups on Monday and Wednesday, counterbalancing for order of the day tested, and balancing participants’ grades and ages. Our recruitment approach was specifically designed to minimize differences due to unknown underlying confounding variables before randomly assigning participants to the testing conditions. This follows the logic of “minimization” or “comparability” of the groups before applying random assignation, rather than Fisher’s traditional automatic, blind group randomization (62). The main reason to use this alternative theoretically-guided criterion before randomizing is that actual randomization is only valid (that is, it assures that significant differences are due to the experimental manipulation rather than to unknown variables intrinsic to the group themselves) with large sample sizes [see mathematical computational and simulation studies reviewed in (63)]. The goal of minimization in the case of studies with small samples such as ours is to increase homogeneity of the participants (by matching a number of basic demographic and individual variables) so that it can be assumed with a degree of confidence that random assignation to testing time was applied on reasonably comparable subsamples and therefore any statistical significant differences were most likely due to the experimental treatment not to intrinsic (unknown or undetermined) differences already present in the groups at the beginning of the experiment [for methodological defense and discussion, see (63)].

Testing took place in a quiet room inside the school library and was scheduled during two ordinary non-consecutive school weeks not including or temporally close to long weekends or holidays (testing was completed in the span of 12 non-consecutive weeks in the Winter school term). School started at 9:15 and ended at 15:00.

**Figure 1:**
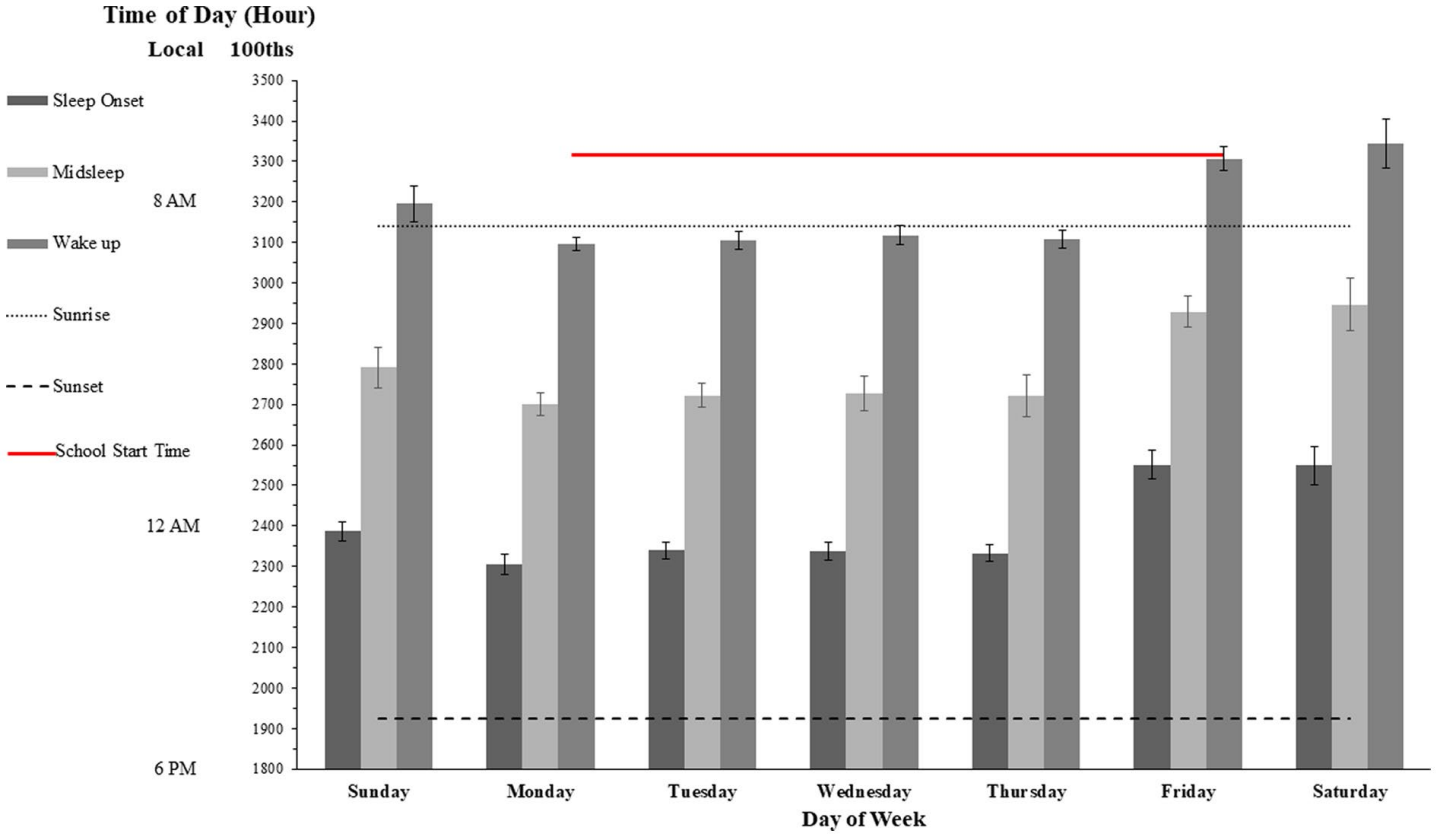
Midsleep and wakeup times for each weekday. Mean midsleep for workday and free day are shown with gray and black dashed lines, respectively. SJL shown calculated to be 123 min. Error bars are SEM. Time of the day is expressed as hundreds of hours over 48-h period, key local times are shown for ease of interpretation; notice that minor ticks correspond to ½ hour (30 min).

Daylight estimates for March and April were derived from data collected by Environment and Climate Change Canada.^2^ The average sunrise for the location and period studied was, in local time, 6.54 [95%CI: 6.35–7.13], whereas average sunset was 19.23 [595 CI: 19:31–19:54]. Therefore, daylight exposure for our sample spanned approximately little over 12 h on average. From Figure 1, it can be extrapolated that during school days participants went to bed and started sleep on average approximately four hours after sunset and woke up approximately 20 min before sunrise.

Since we wanted to focus on achievement-related cognitive performance during school week, and not outside that period, we chose a midweek day as the comparison day to Monday because in the particular school system we conducted the study classes run from Monday to Friday. The sleep phase delay generally and theoretically kicks in on Friday night, but there is no classes or academic testing on Saturdays, so comparing Monday to Friday did not fit our objectives. Finally, Wednesday was chosen over Thursday because of purely logistic constraints since the Stroop sessions were held during the formally scheduled time for the classes from which participants were recruited (i.e., psychology course).

### 2.4. Congruent/incongruent Stroop judgment protocol

We adopted an alternative version of the Stroop –the matching color-word judgment task –often used in cognitive (64–67), developmental (68, 69), and clinical (70) research. The present Stroop protocol was designed to retain as much as possible of the characteristics of the neuropsychological administration for probing cognitive control, however, its primary purpose was to work reasonably well in EEG recording environments [see NeuroScan (71)]. The traditional Stroop color-word task involves responding to the print color of a read word but ignoring its congruent (i.e., BLACK) and incongruent (i.e., RED) meaning, either in wordlist or single-word trials. In the version we used, the standard color-word stimuli are presented, but the task commission is to respond whether color and meaning match. Participants judged the congruency between font color and meaning of Stroop-color words (i.e., congruent: “black”; incongruent: “red”) by pressing one of two predesignated keys on a hand-held pad. Consequently, the motor response demands were generally less than in the traditional versions, reducing the potential contamination of motor response artifacts [see discussion in (69)]. Words were presented for 200 ms followed by 1,000 ms of inter-stimulus interval. Responses deadline was 1,000 ms after stimulus presentation. Words were generated with Stim^2^ software and presented in the center of a 17” Dell LCD monitor (with the following settings: contrast ratio = 400:1, hue = 4,000 K, and luminance = 120 cd/m^2^). The words were capitalized at 18-point size with Arial type font at approximately 2 degrees of visual angle (60 cm from chin rest). The order of presentation was randomized. However, consistent with the so-called Oddball Stroop manipulation featured in hundreds of studies [e.g., (72–74)], the ratio of incongruent (*n* = 224) to congruent words (*n* = 76) was 3:1 in order to counterbalance possible novelty effects (75). Therefore, our task involved relatively *high incongruency* demands and, therefore, relatively high task control demands. The color-word judgment task we used seems appropriate for probing higher-level executive monitoring of the most pertinent response during the task, or “strategic maintenance of the task set” (76) – a complex aggregate of skills also known as cognitive control.

### 2.5. Electrophysiological data acquisition

The EEG was recorded with EEG “quick-caps” with silver chloride electrodes (Neurosoft, Inc., Sterling, United States). Each participant had nine Ag-AgCl electrodes (Pz, P3, P4, Cz, C3, C4, Fz, F3, and F4) applied according to the 10–20 international system. Electrode sites were specifically selected based on previous work (77). All electrodes were referenced to average. Impedances were kept below 5 kOhms. The vertical electrooculograms (VEOG) were recorded from two split bipolar electrodes on the left and right supraorbital ridges (VEOGU, L and R) as well as the left and right zygomatic archs (VEOG, L and R). The signal from the electrodes was amplified and digitized by a SynAmps2 and a SCAN™ 4.3 EEG system (Compumedics Neuroscan, El Paso, TX, United States) with filter settings at 0.15 Hz (high pass) and 100 Hz (low pass). The data were digitized online at a sampling rate of 1,000 Hz. EEG data from four participants were discarded, in three cases due to bad channels during acquisition and in one case because the Stroop task was not completed.

At the beginning and end of the Stroop sessions, for about 5 min, the participants were routinely invited to remain in a state of relaxed wakefulness (trying to minimize blinking and movements) and acclimatize to the setting and wearing the caps. These resting EEGs were used for baseline correction, eye-movement, and artifact reduction modeling calibration and for control analysis on alertness states during the sessions and comparison with the event-locked EEG band power analysis (see below). The experimenter gave the ready signal marking the beginning of the actual experiment (practice phase) whereby the participants could self-initiate the computerized task.

### 2.6. ERP processing

Each participant’s EEG was epoched (100 ms pre-stimulus and 1,000 ms post-stimulus) and averaged with respect to the onset of each word presented, for both congruent and incongruent trials. Ocular artifact reduction was based on a standard automatic regression-based artifact correction through which OEG and eyeblink activity is estimated by linear regression across time and then subtracted from the EEG signal (78). Outliers were defined as EEG epochs exceeding +/− 100 μV threshold and eliminated through automatic artifact rejection. Baseline correction was based on the 100 ms pre-stimulus interval. Only valid trials (with correct responses) were processed and submitted to further analysis.

### 2.7. EEG band-power

Power spectra from 0.5 to 30 Hz were computed by conducting fast Fourier transformation (FFT) on the EEG epochs as defined earlier. The FFT was performed using artifact-free EEG data (1,024 points). The EEG frequency bands were identified as follows: 0.5–3.9 Hz (delta), 4–7.8 Hz (theta), 7.9–12.6 Hz (alpha), 12.7–30 Hz (beta). Given the specific scope of this study (see Introduction), our analysis was narrowed down to the midline frontal electrode Fz.

### 2.8. Data analysis

Sleep logs were used to calculate average fall-asleep time, wake-up time, and total sleep hours, planned paired t-tests were used to examine differences between the means of those three variables comparing weekend vs. weekdays. From these bedtime behavior measures, we derived estimates of week and daily Social Jet Lag (SJL), and sleep deprivation associated with School Start Time (SST-SD). Following the methods by Roenneberg and associates (79), SJL was calculated for the data of the schooldays (Mon-Fri) and week end days (Sat-Sun). Furthermore, we adapted this formula to derive SJL of each workday (Mon-Fri) in the context of the social jetlag for the week. SST-SD was operationally defined as mean residuals of actual hours slept from the mean of expected optimal sleep (i.e., 9 h) as a function of weekday. The latter measure was also used to derive an approximate estimation of daily sleep change throughout the week for supplementary control analyses.

For all other analyses, GLM models were used, through ANOVA or follow-up focused contrasts (80). Reaction Times (RTs) of correct responses and mean accuracy (percent correct responses) were first reduced in terms of Stroop interference measures. Accordingly, we defined Stroop processing speed interference as the difference scores of mean congruent RTs minus mean incongruent RTs. Similarly, we defined Stroop accuracy interference as the difference of mean percentage for accurate congruent matches minus the mean percentage for accurate incongruent matches. These interference scores were submitted to a 2 (*Time* of testing: morning vs. afternoon; Between-subjects) × 2 (*Day* of week: Monday vs. Wednesday; Within-subjects) mixed-model ANOVA. Subsequently, ANOVA-based t-test and F-test contrasts were performed to examine focused *post hoc* comparisons. Within Subjects effects were corrected using the Greenhouse–Geisser adjustment.

The Stroop ERP effect was operationalized by computing *difference waveforms* obtained by subtracting ERPs in the congruent trials from ERPs in the incongruent trials. For each test session, there was only one difference waveform, calculated as the average of all valid congruent ERPs subtracted from the average of all valid incongruent ERPs. The analysis followed standard peak amplitude analysis focused on the time windows of interest (81). Possible offsets in the components’ latencies, were investigated with the *fractional area latency algorithm* (82) by extracting the most accurate measures of the mean mid-point waveform latencies and their distribution within each signature time-window (the time-point dividing the area of the averaged component waveform in half, i.e., 50% of the area under the peak).

In the present study, Stroop-concurrent ERPs from midline frontal (Fz), Central (Cz) and Parietal (Pz) electrodes were examined in correspondence with three well-known waveform time windows which have featured prominently in the literature on cognitive control. These electrodes reflect neurofunctional activity in a midline frontoparietal network known to be associated with cognitive control, which includes, specifically, prefrontal (e.g., Dorsolateral Prefrontal Cortex, DLPFC), midfrontal (e.g., Anterior Cingulate Cortex, ACC; Middle Inferior Frontal Gyrus), motor (Pre-SMA and SMA) and the junction of parietal posterior and extra-striate cortices [see comprehensive review in Carter and Van Veen (49)]. In particular, the P300 waveform (250–400 ms) was here examined as putative marker of initial cognitive evaluation of conflict (64–66). The N450, a waveform with negative amplitude (400–700 ms) was examined for evidence of congruency or incongruency decision and response selection, execution and monitoring (64, 67). Finally, the Sustained Potentials (700–1,000 ms) were considered as typical correlates of post-conflict evaluation and updating/maintenance of task demands strategy set in working memory (68, 69).

The Stroop effect on the EEG power spectra (power Stroop effect) was quantified as the mean absolute power (μV^2^) of incongruent trials minus the mean absolute power of congruent trials.

Finally, we performed a control Fast Fourier Transform analysis (using Brain Electrical Source Analysis, BESA v5.4.28, program routines) on the “resting” segments of continuous EEG that were uncontaminated or corrected for artifacts.

## 3. Results

### 3.1. Sleep data

**Figure 2:**
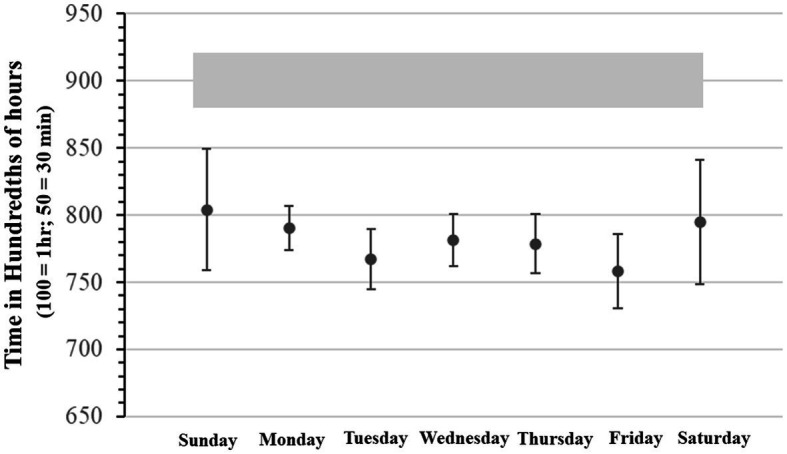
Mean and 99% confidence intervals for sleep logs for each day of the week. The gray box above panel A denotes the 99% confidence interval of the recommended amount of sleep for adolescents [see American Academy of Pediatrics (81)].

The graphs in Figure 2 show means and 99% confidence intervals (CIs) for the total amount of sleep derived from the bedtime logs, when this was calculated as the *total difference between estimated fall asleep time and wake up time (CIs are based on pooled standard deviations of the difference scores)*. The data are represented in the consolidated timeframe of a single week (from Sunday to Saturday) given high intercorrelations between the log self-reports. Median two-week test/retest reliability was *r_s_* = 0.77 (*p* < 0.01). The mean estimated times for turning off the light and being awake the 2 weeks prior testing were highly inter-correlated (median *r* = 0.96) with the estimated times of winding down, falling asleep, getting up during the 2 weeks of testing (all data are plotted together in Supplementary Figure 1). Notably, skewness as measured by kurtosis for all estimates concerning bedtimes, and especially “winding down” was between-1 and-2 (negative skew), which means that the bulk of the observations making up the distributions corresponding to all those means laid systematically left or above the means. This implies that most of the students in the group had extended wakefulness during evenings relative to the estimated means (whose grand mean is ~11:30 pm; thus, most students were typically in bed and/or asleep past this time of reference), therefore supporting the assumption of an evening preference chronotype for most of the participants (there were no reliable statistical differences between the Morning and Afternoon subsamples). There was no significant skewness for the distributions of awake and getting up times. For all variables kurtosis was below 1.4, indicating that the parametric statistical procedures we used for all other analysis were sufficiently robust to handle the observed normality violation.

Over the 2 weeks the mean reported sleep total hours and minutes was 7 h and 47 m (SD =122 min), regardless of whether it was the weekend or school night, there was no significant difference in total amount of sleep. However, the estimated 99% confidence interval in overall week school sleep duration for our sample was between 6.98 h (i.e., 6 h and 59 min) and 8.62 h (8 h and 37 min). Considering the corresponding sampling distribution and using the estimated means from the American Academy of Pediatrics (83) the rough estimated amount of sleep in terms of 99% population confidence interval recommended for this population of adolescents could be estimated between 8.8 h (8 h and 48 min) and 9.2 h (9 h and 12 min). Thus, according to this estimation, given that the observed confidence intervals were non-overlapping, the amounts of total hours slept were significantly below the estimated recommended quota during the entire week. Visual comparison between the population confidence interval of recommended levels of sleep versus observed sleep hours levels reported by our sample suggests a differential gap which possibly indicates some chronic loss of sleep during the entire week.

On weekdays, the mean reported fall asleep proxy was 11:28 pm (SD = 70 m). The mean reported wake-times was 7:35 am (SD = 96 m). However, on weekends, the mean reported fall asleep times were 1:30 am (SD = 139 m) for Friday night and 1:29 am (SD = 106 m) for Saturday night. The mean reported wake-up times were 9:05 am (SD = 81 m) for Saturday morning, and 9:26 am (SD = 136 m) for Sunday Morning (see Figure 2, Panel B). Therefore, the average pattern of fall-asleep vs. wake time over the 2 weeks showed a significant delay of over 2 h (*t*(23) = 2.99, *p* < 0.05).

Although all asleep time for Sunday night was slightly later than the average sleep time during the week (around 11:45 pm, as opposed to around 11 pm) the wake-times for Monday morning were also later than the rest of the week (~ 8 am, SD = 57 min, as opposed to ~7:10 am, SD = 28 min), this delay was significant (*t*(23) = 2.95, *p* < 0.05). Thus, although the 45–60 min delay in waking time on Monday could be a compensatory response to the delayed week end bedtime to maintain a constant sleep duration, the previous analysis suggests that there was also a modest sleep deprivation as compared to later days of the week.

Mean ratings of quality of sleep increased modestly but significantly with week progression (F (1, 17) = 4.75; *p* < 0.05) while the increase in mean ratings of alertness during the week was not significant (*F*(1, 18) = 3.00; *p* = 0.10). Data for both variables are shown in Supplementary Table 2.

### 3.2. Mid-sleep, week and daily social jet lag, and school start time sleep deprivation

Figure 1 shows mean sleep onset, wake up and *mid sleep* times calculated as in Roenneberg et al. (79) but expressed in the 48-h cycle from Sunday to Saturday. The Figure also displays the average sunrise and sunset for the period studied. Social jetlag calculated for the week using Jankowski’s sleep-corrected formula [i.e., absolute value of sleep onset on week end minus sleep onset on school days, Jankowski (84)] was determined to be an average of ~123 min (i.e., 2 h and 3 min), which is consistent with adolescents’ estimates in a substantial body of research (85). This estimate is also virtually identical to the delayed sleep onset estimated by total amounts of sleep reported above. Indeed, SJL was closest to 0 min on Sunday, indicating that the ‘correction’ made for weekly jetlag on Saturday produced a balancing effect that produced optimal sleep patterning on Sunday. Differently than for the total sleep time, mid-sleep focused contrasts revealed that on both Monday and Wednesday the participants slept significantly less than on Sunday (*t*(23) = 5.59; *p* < 0.001), and that they slept less on Monday than on Wednesday although this difference was only marginally significant (*t*(23) = 1.84; *p* = 0.078). We note here that we call these comparisons “focused contrasts,” traditionally known also as “planned contrasts or t-tests,” exactly because following the main hypotheses we only examined comparisons between Monday Wednesday and weekend. Also notice that although in this case multiple comparison adjustment is not needed, the comparison between weekend and the other 2 weeks periods yielded rather robust effects which survive even the most conservative Bonferroni correction.

**Figure 3:**
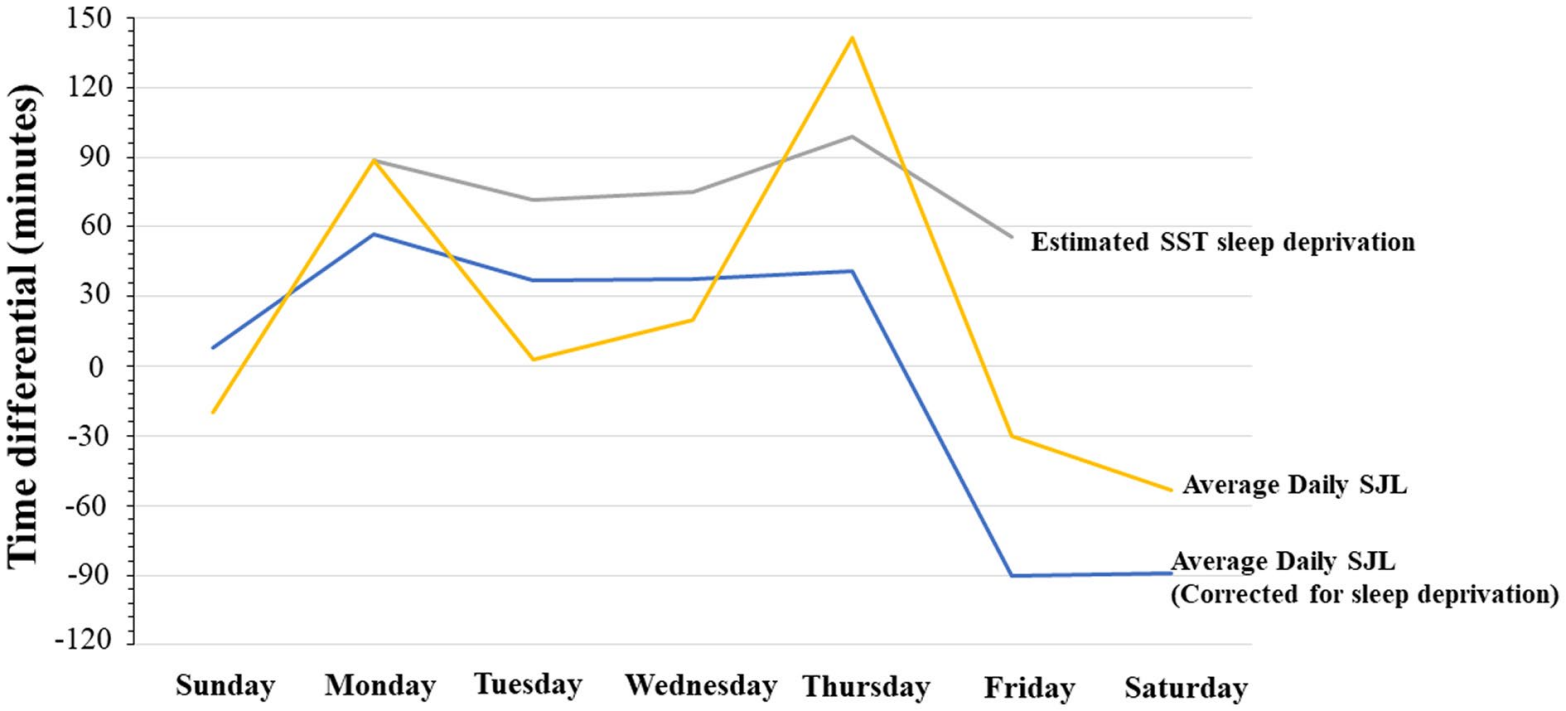
Average Daily Social Jet Lag, Average Social Jet Lag corrected for sleep deprivation, and average daily sleep deprivation related to school start time. All expressed in minutes.

SJL was also determined daily in two ways, first by deriving the all-inclusive uncorrected SJL, with an adaptation of the formulas used by Roenneberg et al. (79). Second, by deriving the sleep-deprivation corrected SJL using Jankowski’s formula (by subtracting each day’s average sleep onset from average weekend sleep onset and scaled as a function of 2 work/school weeks). Furthermore, School Start Time sleep deprivation (SST-SD) was estimated by taking *the difference between the mean of daily expected optimal sleep (i.e., 9 h) and actual daily hours slept*. These measures are shown together in Figure 3. The comparison between the sleep-deprivation corrected SJL and the uncorrected SJL shows that SST-SD seems to interact differently during the school week. That is, SST-SD interacts additively with SJL on Monday, by contributing more sleep loss to SJL, but it interacts with it antagonistically or subtractively on Wednesday, as the SJL effect drops below the level attributable to the SST contribution. We report these differences as calculated on single estimation average points, as done in other studies which instead use longer continuous records, because they only serve as qualitative trend estimates that can be cross-referenced with our other objective measures. It is worth noticing that in Figure 3 the standard deviation of SJL with or without sleep deprivation correction, was only 37 and 45 min, respectively. When compared to the overall change in values – ranging over more than 240 min (i.e., 4 h) – it can be seen that in all cases variance could be at most roughly 1/5th of the entire distributions, indicating that we did not obtain particularly extreme values requiring the supplementary application of smoothing techniques.

### 3.3. Behavioral data

#### 3.3.1. Reaction time interference

**Figure 4:**
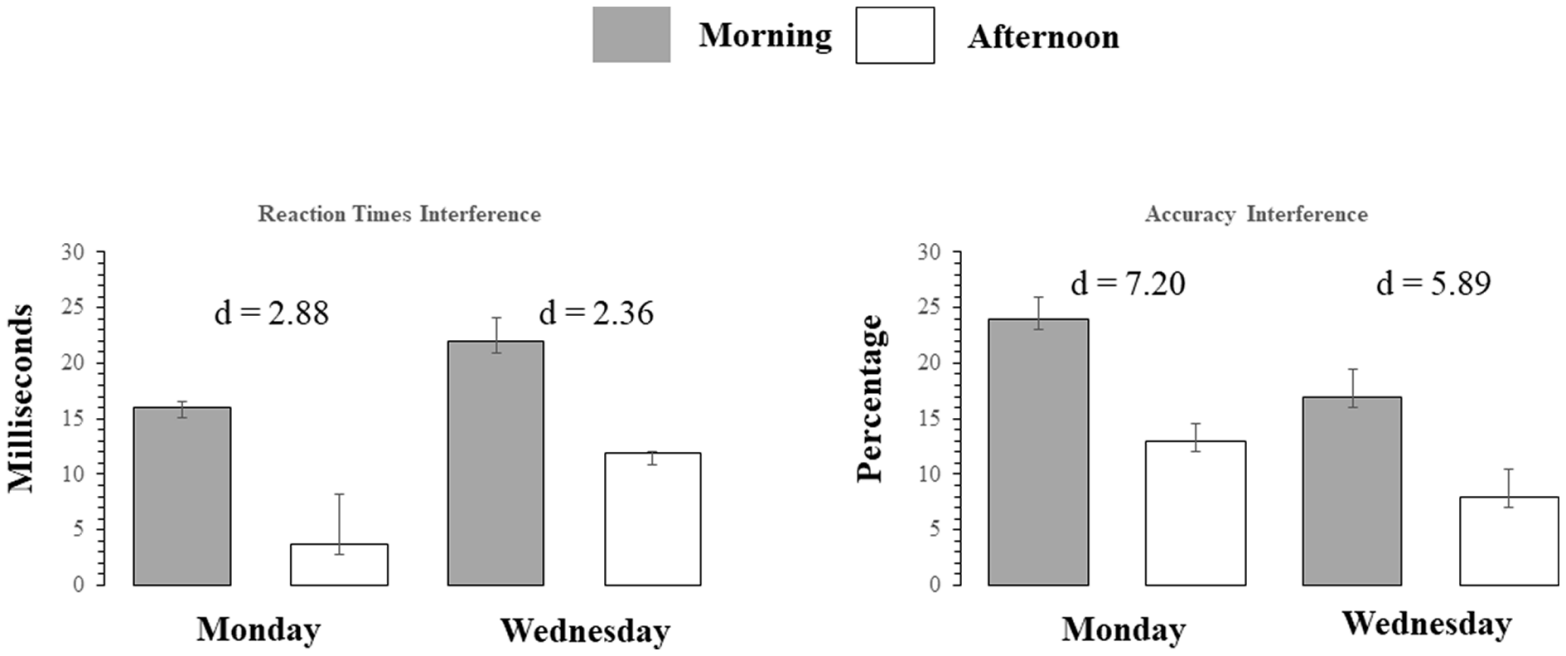
Left Panel: Reaction time Interference for correctly answered trials, for each Stroop trial type on Monday and Wednesday (AM and PM) testing. Right Panel: Accuracy interference in percent correct, for each Stroop trial type., on Monday and Wednesday (AM and PM) testing. Bars indicate standard errors.

Reaction Times and the Stroop interference differences for correct responses across all congruent and incongruent trials are displayed in Table 1. The effect of Stroop interference on RTs (difference scores defined as: mean incongruent RTs minus mean congruent RTs) is also highlighted graphically in the left Panel of Figure 4 which also displays the Cohen’s d coefficients for the AM-*vs*-PM effects. The Week-day X time of day interaction was significant (*F*(1, 23) = 40.22, *p* < 0.001) as was the main effect of time of day (*F*(1,23) = 32.70, *p* < 0.001), showing that interference was always larger in the morning than in the afternoon. However, the main effect of day of week was not significant (*F* < 1).

**Table 1:** Mean percent of accuracy, mean reaction times, and interferences (with ±1 standard errors) for Congruent and Incongruent trials in a Stroop color-word matching task by female high-school students.

Time
		Day				
	Monday			Wednesday	

	*M*	*S.E.*		*M*	*S.E.*

	*Accuracy*				
Morning
Congruent	58%	3.0%		61%	3.0%
Incongruent	82%	1.0%		78%	2.0%
Interference	24%	2.0%		17%	2.5%
Afternoon
Congruent	75%	2.0%		75%	2.0%
Incongruent	88%	1.0%		83%	3.0%
Interference	13%	1.5%		8%	2.5%

	*Reaction Times(ms)*				
Morning
Congruent	524.51	13.34		545.72	8.49
Incongruent	540.60	13.76		567.70	6.33
Interference	16.08	0.42		21.98	2.17
Afternoon
Congruent	551.21	13.39		528.16	4.95
Incongruent	554.95	17.84		540.03	4.77
Interference	3.74	4.45		11.88	0.18

The main and interaction effects are dissected in Table 2, which presents t-contrasts based on a One way repeated measures ANOVA. The contrasts show clearly that RT interference on Monday morning did not differ significantly (as per Bonferroni-adjusted threshold) from Wednesday afternoon, whereas interference on Monday afternoon was significantly smaller than Wednesday morning. The interaction was therefore driven by the fact that the interference on Monday afternoon was very small. When one considers the absolute effect size of the morning vs. afternoon effect, as estimated by Cohen’s d indicator [2.88 (Monday) vs. 2.36 (Wednesday)], it is possible to observe that there was only a slight difference and the effect size of time of day was similar. This indicates that the effect size difference is consistent with the interpretation of additive interaction between SJL and SST-SD as shown in Figure 3.

**Table 2:** Stroop interference on mean accuracy and mean reaction times compared by day of school week and time of day.

Contrast	RT interference (in ms)		Accuracy Interference (in %)		
	Difference	*t* (23)	Difference	*t* (23)
MoA vs. MoP	12.34	7.05*	11.00	17.63*
MoA vs. WeA	−5.90	−3.37	7.00	11.23*
MoA vs. WeP	4.20	2.40	16.00	25.64*
MoP vs. WeA	18.24	10.42*	−4.00	−6.41*
MoP vs. WeP	8.14	4.65*	5.00	8.01*
WeA vs. WeP	10.10	5.77*	9.00	14.42*

MoA, Monday Morning (AM); MoP, Monday Afternoon (PM); WeA, Wednesday Morning (AM); WeP, Wednesday Afternoon (PM).

Bonferroni threshold *p* < 0.002.

#### 3.3.2. Accuracy interference

Mean accuracy (% correct response) calculated for each testing time and day for both congruent and incongruent trials and accuracy interference are shown in Table 1. The effect of Stroop interference on accuracy (difference scores defined as: mean incongruent accuracy minus mean congruent accuracy) is also highlighted graphically in the right Panel of Figure 4 with the Cohen’s d coefficients for the AM-*vs*-PM effects. Main effects of week day and time of day, and their interaction were all significant (all *F*(23)’s > 59.47, *p* < 0.001). Main and interaction effects are dissected in Table 2, which presents t-contrasts based on a One-way repeated measures ANOVA. The contrasts show clearly that RT interference on Monday morning differed significantly and was quite substantially larger (as per Bonferroni-adjusted threshold) than Wednesday afternoon, whereas interference on Monday afternoon was significantly smaller than Wednesday morning, with effect going in diametrically opposite direction than the previous test for the RT interference interaction. The interaction was therefore driven by the fact that not only interference was of substantially discrepant relative magnitudes but also in opposite directions (i.e., they “crossed”). When one considers the absolute effect size of the morning vs. afternoon effect, as estimated by Cohen’s d indicator [7.20 (Monday) vs. 5.89 (Wednesday)], it is possible to observe that there was a substantial difference and the effect size of time of day was much smaller on Wednesday than on Monday. This indicates that the effect size difference is consistent with the interpretation of subtractive interaction between SJL and SST-SD as shown in Figure 3, which seems explained by the mid-week decline in SJL.

### 3.4. Stroop ERP difference waveforms (d-waves)

Initially, we conducted the customary inspection of ERP grand averaged data by visually identifying peaks (components) within approximate millisecond latency ranges that defined landmark signatures: *Positive 300 ms-complex* (P300), *Negative 450 ms-complex* (N450) and *Sustained Potentials* (SP) overlapping with response (86). The identification of ERP components was validated by contrasting averaged waveforms from all conditions and comparing the differences simultaneously by using a bin-by-bin automatic (“blind”) peak amplitude analysis procedure whereby, the effects were quantified via standardized differences (i.e., focused contrasts corrected for multiple comparisons) across the entire averaged epochs’ sweep (87).

**Figure 5:**
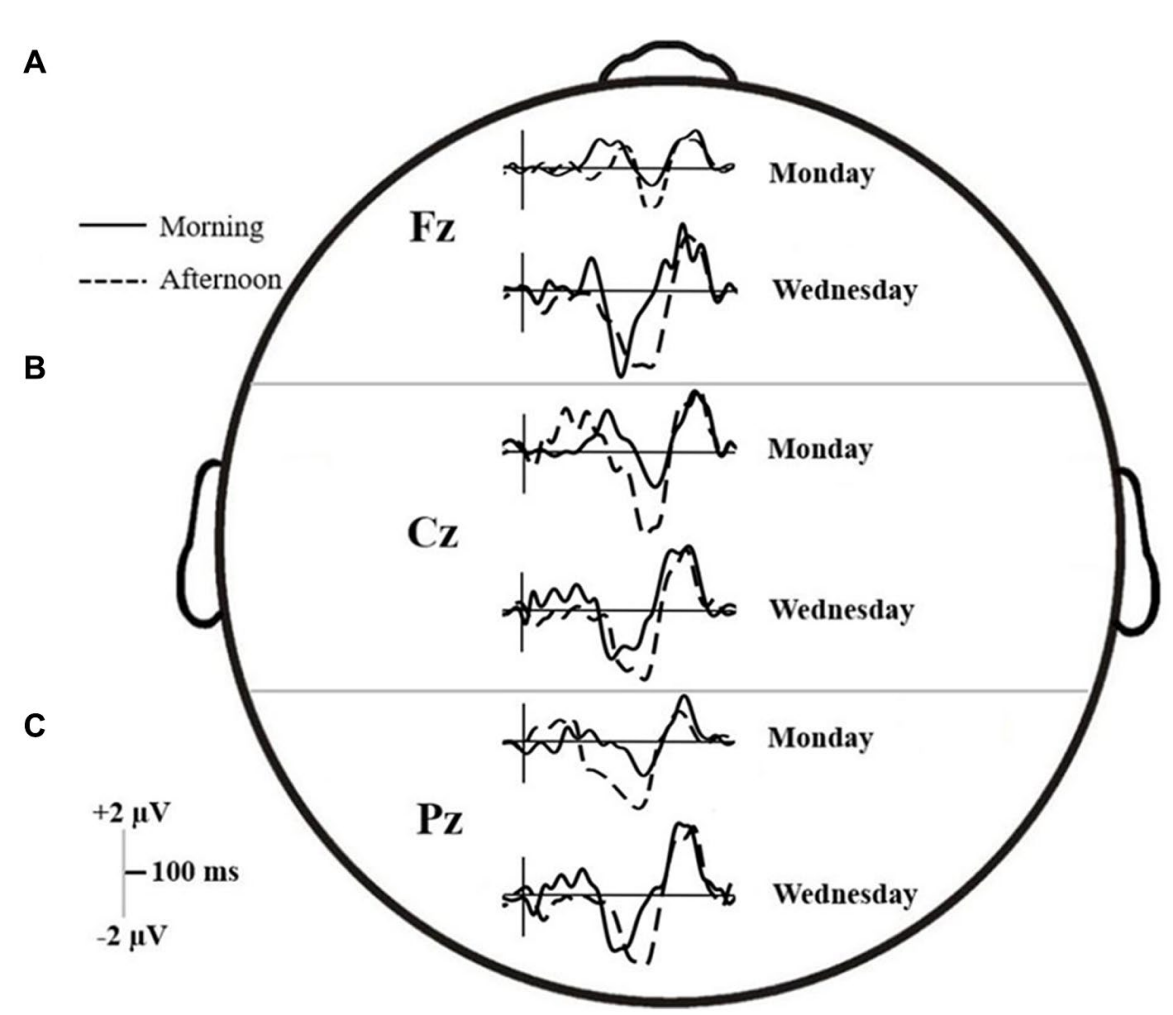
ERP trace for the **(A)** Fz, **(B)** Cz, and **(C)** Pz electrodes on Monday and Wednesday, in both the morning (solid line) and afternoon (dashed line) testing times.

To reduce the interaction order to 2-way, the Stroop ERP effect was quantified by computing the amplitudes of difference waveforms (henceforth *d-waves*) obtained by subtracting ERPs in the congruent trials from ERPs in the incongruent trials. Note that although using these types of difference scores is widely accepted and used practice in Stroop research (75), the meaning of the direction of the polarity (i.e., positive or negative) can only be interpreted by reference to the original known ERP waveforms. The latter approach was adopted in all analysis reported below. Coherent with our objectives (see Introduction), the analysis focused specifically on the three midline electrodes: Fz, Cz, and Pz; the d-waves for these electrodes are shown in Figure 5.

#### 3.4.1. Amplitude data

Time series analysis of the individual and group averaged amplitudes of d-wave data points (1,100 data points per group) was further simplified by using same-density bins of 100 ms (200 sampled grand-averaged waveform data points per bin). These bins were determined for the entire epoch (−100 to 1,000 ms) and the averaged amplitudes were assessed separately for the three electrodes. Accordingly, grand-averaged d-waves were computed separately by Time × Day conditions and partitioned into six time intervals defining six components representing relative differentials in the corresponding landmark signatures: Early potentials (*EP*:100–199 ms), P300 (200–399 ms), Early N450 (400–599 ms), Late N450 (600–699 ms), Early SPs (700–849 ms) Late SPs (850–999 ms). Binned participants’ amplitude averages were then aggregated in binned grand averages split by conditions. ANOVA contrasts were based on the binned data.

Across the six time intervals, there were no interactions, but both main effects of Time and Day on d-waves amplitudes were significant. The mean differences and results of the contrasts are summarized in Table 3. At the frontal midline electrode (Fz), amplitudes were significantly higher (either positive or negative polarity) on Wednesday than Monday in all windows of interest except EP and early N450. In addition, P300, Early N450 and Late SP-related d-waves all showed higher and relatively more positive amplitudes in the morning than in the afternoon. Similarly, the late N450-related d-waves showed higher negative amplitude in the afternoon (see Panel A of Figure 5). At the central midline electrode (Cz), d-waves in the afternoon showed higher amplitudes in both N450-related windows, they also showed much higher amplitudes on Wednesday in late N450 and both early and late SP-related windows (see Panel B of Figure 5). Finally, at the parietal midline electrode (Pz), there were no reliable significant differences between morning and afternoon, however, d-waves amplitude corresponding to late N450 and both SP windows showed higher negative amplitudes on Wednesday (see Panel C of Figure 5). In sum, across the midline generally much greater activity was observed at the early stages of processing and then overlapping with response on Monday and in the morning, whereas much greater activity was observed in later processing before response on Wednesday and in the afternoon.

**Table 3:** Mean differences and ANOVA-based focused contrasts for Stroop ERP amplitudes of difference waves compared over Time (morning vs. afternoon) and day (Monday vs. Wednesday) of testing.

		Source of effect					
		Time^a^		Day^b^		Time × Day	
		*M diff.*	*F*	*M diff.*	*F*	*M diff.*	*F*
Difference ERP wave testing	

			(Electrode Fz)				
EP		0.15	< 1	0.14	< 1	0.89	2.30
P300		0.67	11.31*	−2.53	39.88*	0.82	1.94
N450	Early	0.93	21.71*	−0.55	1.87	1.38	5.50
	Late	−0.89	19.78*	−2.37	34.79*	1.57	7.16
SP	Early	−0.28	2.00	−2.86	50.79*	0.78	1.78
	Late	0.94	22.24*	−3.99	99.15*	0.51	< 1

			(Electrode Cz)				
EP		−0.38	< 1	−1.82	8.98	2.48	4.52
P300		1.03	6.81	−1.05	2.98	1.93	2.72
N450	Early	−1.68	17.97*	0.28	< 1	0.80	< 1
	Late	−1.36	11.79*	−3.33	30.11*	0.97	< 1
SP	Early	−1.25	9.92	−8.33	188.50*	0.32	< 1
	Late	−0.67	2.88	6.07	99.97*	0.06	< 1

			(Electrode Pz)				
EP		−0.90	7.74	−0.84	1.83	0.00	< 1
P300		0.93	8.27	−0.25	< 1	1.12	1.38
N450	Early	−0.82	6.44	0.34	< 1	0.49	< 1
	Late	−0.25	< 1	−3.00	23.53*	1.78	3.46
SP	Early	0.64	3.95	−10.94	312.15*	1.54	2.59
	Late	0.22	< 1	5.00	65.09*	2.19	5.26

Values represent ERP difference waveform amplitudes measured in microvolts (μV). M diff. = mean difference. EP: Early Potentials (100–199 ms), P300 (200–399 ms), Early N450 (400–599 ms), Late N450 (600–699 ms), Early SPs (700–849 ms) Late SPs (850–999 ms). ^a^A positive sign indicates higher amplitude for the morning d-wave; a negative sign indicates a higher amplitude for the afternoon d-wave. ^b^A positive sign indicates a higher amplitude for the Monday d-wave; a negative sign indicates a higher amplitude for the Wednesday d-wave.*p ≤ 0.0025; Bonferroni corrected. Dfs = 1.19.

#### 3.4.2. Latency data

The extracted 50%-peak latencies were analyzed in the same way as the binned amplitudes. The results are summarized in Table 4.

**Table 4:** Mean differences and ANOVA-based focused contrasts for Stroop ERP median time-latencies of difference waves compared over Time (morning vs. afternoon) and day (Monday vs. Wednesday) of testing.

		Source of effect					
		Time^a^		Day^b^		Time × Day	
		*M diff.*	*F*	*M diff.*	*F*	*M diff.*	*F*
Difference ERP wave	

			(Electrode Fz)				
EP		−1.62	< 1	−9.75	10.16	12.75	8.69
P300		−0.72	< 1	66.75	476.22*	1.50	< 1
N450	Early	5.76	9.15	38.63	159.46*	15.00	12.02*
	Late	10.80	32.18*	28.50	86.82*	19.50	20.32*
SP	Early	−6.84	12.91*	−75.75	613.30*	43.50	101.12*
	Late	8.64	20.60*	−14.25	21.70*	12.00	7.70

			(Electrode Cz)				
EP		1.80	< 1	−2.25	< 1	7.50	< 1
P300		1.80	< 1	4.50	< 1	18.00	4.42
N450	Early	5.40	8.23	33.00	29.73*	28.50	11.09
	Late	−13.68	52.82*	24.00	15.72*	58.50	46.71*
SP	Early	10.98	34.03*	27.38	20.46*	140.25	268.48*
	Late	1.44	< 1	−3.75	< 1	9.00	1.11

			(Electrode Pz)				
EP		0.72	< 1	5.25	< 1	3.00	< 1
P300		2.88	2.33	1.13	< 1	27.00	12.06*
N450	Early	6.30	11.15	35.63	41.98*	65.25	70.42*
	Late	−7.02	13.85*	25.50	21.51*	33.75	18.84*
SP	Early	33.66	318.40*	56.63	106.06*	27.75	12.74*
	Late	−0.54	< 1	1.12	< 1	2.25	< 1

Values represent median ERP difference waveform latencies measured in milliseconds (ms). M diff. = mean difference. EP: Early Potentials (100–199 ms), P300 (200–399 ms), Early N450 (400–599 ms), Late N450 (600–699 ms), Early SPs (700–849 ms) Late SPs (850–999 ms). ^a^A positive sign indicates a latency delay in the morning d-wave; a negative sign indicates a latency delay in the afternoon d-wave. ^b^A positive sign indicates a latency delay in the Monday d-wave; a negative sign indicates a latency delay in the Wednesday d-wave. *p ≤ 0.0025; Bonferroni corrected. Df = 1.19.

In all electrodes, interaction Time × Day effects on latencies were found for late N450 and early SP windows. Interaction effects related to the early N450 were also found at Fz and Pz, but not at Cz (which however was marginally significant). For the most part, the statistical analysis confirms a similar pattern that can be discerned by comparing Panel A and C, referring to each electrode in Figure 5. N450-related and early SP-related d-waves reached 50% of their maximum amplitude significantly faster on Wednesday than on Monday. However, while there were no latency offsets associated with testing time on Monday, d-waves were generally delayed on Wednesday afternoon relative to Wednesday morning. A P300-related interaction was also found at Pz, which can be interpreted as showing that on Monday d-waves peaked faster in the afternoon than in the morning, but no such a difference occurred on Wednesday (see Figure 5). Finally, Fz d-waves showed isolated main effects that cannot be accounted by the reported interactions, namely, delayed latencies in the P300 window on Monday, and in the late SP window in the afternoon.

### 3.5. EEG power data

Stroop Event-Related EEG generally showed a range of maximum average activity between 35 and 45 μV for delta, 25–30 μV for theta, 15–20 μV for alpha, and 9–10 μV for beta.

**Figure 6:**
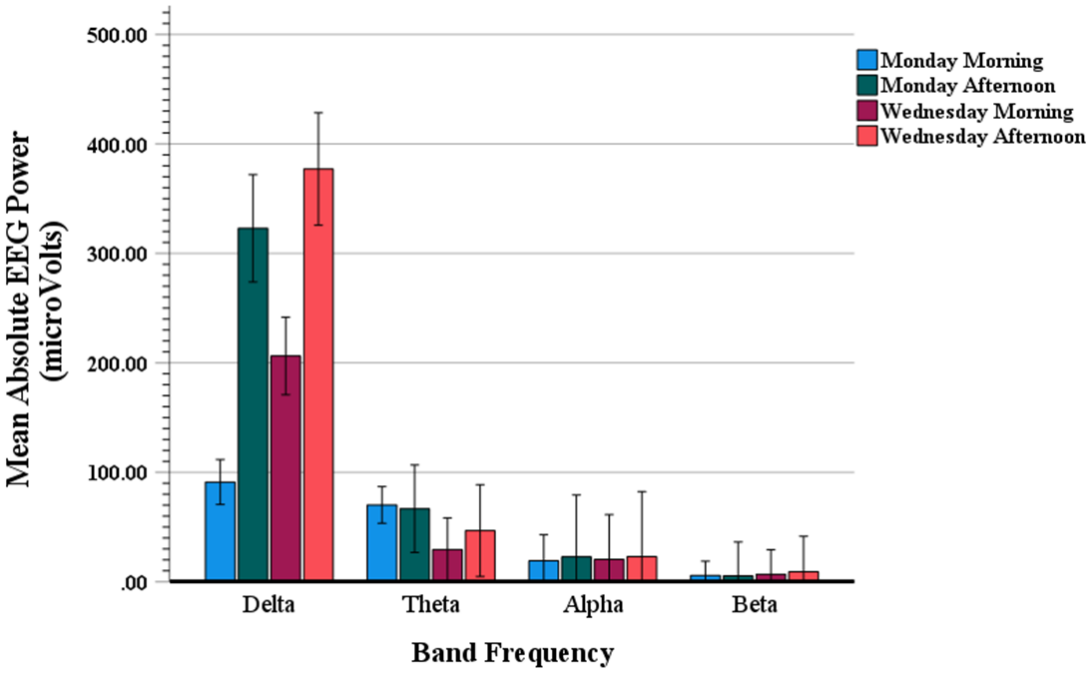
Mean absolute value of Stroop event-related EEG power for delta, theta, alpha and beta band frequencies on Monday morning, Monday afternoon, Wednesday morning, and Wednesday afternoon.

Figure 6 shows mean absolute values of the Stroop effect on the EEG power spectra (power Stroop effect) quantified as the mean absolute power (μV^2^) of incongruent trials minus the mean absolute power of congruent trials. These event-related EEG band power (ERBP) amplitudes are displayed for delta, theta, alpha and beta frequency bands as a function of weekday and time of day testing. An ANOVA contrast analysis with frequency band and testing time conditions (i.e., Monday AM, Monday PM, Wednesday AM, Wednesday PM) yielded a significant interaction (*F*(3,19) = 4.59; *p* = 0.01), with main effect of frequency band also significant (*F*(3,19) = 92.43; *p* < 0.001). Confirming what can be observed in Figure 6, these results indicate that there was a preponderant increase in delta power, and somewhat in theta power as well, as compared to all other frequency bands.

Table 5 shows the results of *post hoc* contrasts with Simes-Bonferroni correction for multiple comparisons. The results show that delta power was significantly higher than any other band frequency except theta just on Monday morning. Also, on both morning and afternoon of Monday, theta was marginally significantly higher than alpha and higher than beta.

**Table 5:** *Post hoc t*-contrasts for mean EEG Stroop-related power differences by day of school week (Monday vs. Wednesday) X time of day testing (AM vs. PM).

	Contrast		Mean Power difference
Monday Morning	Delta vs.	Theta	20.90
		Alpha	71.86***
		Beta	85.33***
	Theta vs.	Alpha	50.96*
		Beta	64.42*
	Alpha vs.	Beta	13.46
Monday Afternoon	Delta vs.	Theta	256.22
		Alpha	300.13
		Beta	317.51
	Theta vs.	Alpha	43.90*
		Beta	61.29**
	Alpha vs.	Beta	17.38
Wednesday Morning	Delta vs.	Theta	176.87****
		Alpha	185.80****
		Beta	199.43****
	Theta vs.	Alpha	8.93
		Beta	22.56
	Alpha vs.	Beta	13.63
Wednesday Afternoon	Delta vs.	Theta	330.27****
		Alpha	354.04****
		Beta	367.94****
	Theta vs.	Alpha	23.77
		Beta	37.67
	Alpha vs.	Beta	13.90

Simes-Bonferroni adjusted significance levels: *p < 0.10, **p < 0.05, ***p < 0.01, ***p < 0.001.

Furthermore, while all other frequencies were similarly distributed across the testing times, the delta amplitudes varied systematically as a function of weekday and time of day. Specifically, the ANOVA-based *post hoc* paired t-contrasts showed a pattern of differences consistent with the trend shown in Figure 6, which are detailed in Tables 5, 6; *t*-contrasts for all other frequencies yielded no significance (all *t*(19)’s < 1.65). The pattern of results for delta reveals that EEG power related to Stroop trials is consistently elevated on the afternoons of both Monday and Wednesday, this outcome is the mirror inverse of that observed for Stroop interference on RTs and accuracy.

**Table 6:** Pairwise ANOVA *post hoc t*-contrasts for delta frequency band.

Contrast
	Mean difference	*t* (19)
MoAM vs.
	MoPM	−231.81	−9.42*	WeAM
		−115.18	−4.68*	WePM
−285.94		−11.61*
MoPM vs.
	WeAM	116.63	4.74*	WePM
−54.13		−2.20
WeAM vs.	WePM	−170.76	−6.94*

MoA, Monday Morning (AM); MoP, Monday Afternoon (PM); WeA, Wednesday Morning (AM); WeP, Wednesday Afternoon (PM).

MSE, 24.62; *Bonferroni threshold *p* < 0.002.

On average, resting EEG data generally showed much smaller amplitudes than ERBPs (with maximum amplitudes within the bounds of 15 ± 8 μV), however the spectral distributions of the frequency bands for Monday and Wednesday and for morning and afternoon were like those observed in the ERBP data in the respective weekdays and times of day testing (Pearson intra-correlations for frequency band group data between resting and Stroop recordings ranged between 0.63 and 0.87, *p* < 0.01). This indicates that the pattern of fluctuations in weekly alertness levels and neurocognitive performance co-varied.

## 4. Discussion

In the present study, female high school students reported approximately 2 h of sleep pattern delay on the weekend, which is consistent with the late-night chronotype generally observed in adolescents (58, 88, 89). Also consistent with previous findings (59), the students reported sleep patterns indicating a moderate weekly cumulative sleep deprivation which co-varied with SSTs, since the peak of the sleep also shifted with the onset of the weekend when SST sleep pressure was temporarily suspended, and sleep duration (see Figure 3) and quality increased (see Supplementary Table 2). We hypothesized that a cooperative effect of SST-induced sleep deprivation accumulation across the week, combined with SJL-induced adjustment at the beginning of the week, would produce a distinct pattern of cognitive variation, as probed by behavioral and electrophysiological measures.

The performance results and ERP patterns for Monday and Wednesday morning and Monday afternoon support the conclusion of an interaction between influences of social jet lag and sleep deprivation related independently to school start times. Although as predicted they were still pronounced in amplitude, ERPs delay on Wednesday afternoon would seem to be somewhat inconsistent with our hypothesis. Below we provide a full step-by-step dissection of the findings, including some limitations of the present study. We argue that the most plausible and parsimonious account for the whole pattern of results is a complex interplay of circadian misalignment (SJL), sleep adjustment occurring on Mondays in response to early SST, and mental fatigue in mid-week afternoons as shown by prominence of high-amplitude delta EEG frequency (and consistent with our explanation for the ERP delay in Wednesday afternoon).

### 4.1. Accuracy and reaction time Stroop interference

As predicted, Stroop interference for both accuracy and RTs was higher in the morning than in the afternoon. However, overall, while RTs tended to be faster on Monday than Wednesday, accuracy was higher on Wednesday than Monday. In addition, the effect size for the difference between morning vs. afternoon was larger on Monday than on Wednesday, in relation to both RTs and accuracy, although the effect size discrepancy was clear and strong for accuracy but only slight for RTs. The latter results show the effect sizes differences reflected variations in the interaction between SJL and SST relative to the two behavioral measures of cognitive performance. While the effect size differences for accuracy reflected the time course of the declining trend of the daily SJL from early to mid-week school days, the effect size differences for RTs seemed rather similar from early to mid-week, suggesting an additional contribution of SST. Thus, the findings support the conclusion that SJL showed a minor association with processing speed, with SST contribution, but it was instead strongly associated with cognitive performance accuracy.

### 4.2. ERPs

Monday afternoons had increased ERP amplitude in the afternoon compared to Monday morning in the P300 window at the frontal midline electrode. Larger negative amplitudes were also observed in the N450 window in the afternoons compared to the mornings across all midline electrodes. Increased amplitude of d-waves in the P300 window suggests increased alertness and cognitive attentional deployment in the afternoon compared to the morning (60, 64–66). The larger N450 d-waves may also indicate that more cognitive processing was involved in the afternoon, since N450 amplitude is modulated by the degree of difficulty, or conflict, of the task. The higher neural activity, as indicated by the elevated N450-related amplitudes observed in the afternoon, could indicate that adolescents perform better in the afternoon when circadian arousal is heightened but at a cost of additional cognitive load (15).

#### 4.2.1. P300 and N450

As compared to Monday, Wednesday d-waves in the P300 window occurred up to approximately 1/10^th^ of a second earlier irrespective of testing time. Wednesday morning appeared to have higher amplitudes in the P300 window compared to afternoon. In contrast, the amplitude in the N450 window was comparable over testing times between testing days; however, Wednesday afternoon N450 and early SP peaks occurred, on average, later than their Wednesday morning counterparts.

#### 4.2.2. Delta activity

The spectral analysis showed that Stroop-related EEGs seem to include a significant proportion of slow waves (delta) typically seen during sleep, especially in the afternoon. Whereas, on Monday, EEG activity showed a mixture of beta and theta oscillations which typically in the midline correlate with sustained cognitive effort and load (62, 90). Wednesday afternoon had the highest mean absolute power for the delta frequency followed by Monday afternoon, Wednesday morning, and Monday morning. The control resting EEG analysis showed that this pattern was not linked just with the task, the spectral patterns of resting activity were correlated with the one observed during the task, although the two differed in terms of intensity of activity. Thus, the observed pattern is inconsistent with a typical diurnal increase in the EEG activity between 1 to 4.5 Hz (91).

One explanation for the high delta activity is that the adolescents may not have adjusted to the SSTs; which may be the case even though our observed SSTs may not be defined as “early” by current pediatric guidelines or school practices. Indeed, morning testing was relatively late (~9:30–10:30) and it seems that may no longer adequately represent early school start (9:30 is later than even the most generous SSTs proposals in the US). For example, Carskadon et al. (43) showed that the profound sleepiness at 8:30 pm observed in a subsample of 10th grade students from starting school early was eliminated by 10:30. Because the present was a study attempting to investigate natural patterns, no sleep schedule was assigned prior to investigation to stabilize the circadian timing of the participants. However, our analysis of the two-week logs prior and during testing showed that our participants, as a group, had a late-night bed preference (estimated >11:30 pm), and levels of alertness as revealed by the resting EEG data correlated with the neurocognitive pattern reflected by the Stroop performance. Thus, there is enough converging evidence in our data to indicate that the circadian timing system of most of these adolescents was misaligned with the timing of school activities on Monday, all day, and Wednesday morning. Since we found no alpha or beta changes, and consistently larger proportions of delta and theta frequencies on Monday and just delta on Wednesday, we suggest that, similar to prolonged wakefulness, while the combined delta theta EEG activity observed on Monday may reflect heightened sensitivity to both error detection and conflict, respectively (92–94), the major elevation in delta on Wednesday may reflect increased error monitoring associated with mental fatigue from cumulative sleep deprivation (95–98).

#### 4.2.3. ERPs and ERBPs

Both past resting EEG research and our ERBP findings focused on changes related to the same underlying neurocognitive state: wakefulness. The link between our patterns of results and the classical findings on resting EEGs in the literature reviewed would seem the more justified by the finding that our control analysis revealed basic qualitative similarities between resting EEG and ERB spectral distributions. That is, for each respective condition, the power of resting EEGs and ERBPs appeared to correlate. In a nutshell, these findings mainly support the interpretation that alpha desynchronization and slow EEG oscillations were relatively more prevalent than expected early in the week during *both* cognitive engagement and rest. It is plausible to assume that ERBPs and resting EEGs reflected some core functional continuity for day and time of testing. The key difference between engaged wakefulness and resting wakefulness, however, concerned the amplitude of power, being far greater during the Stoop task. This is compatible with the well-established bidirectional dynamics between the ascending reticular activating system (ARAS) and the cortex in regulating wakefulness levels along a gradient of neural excitability (99) as well as the emerging evidence that changes in wakefulness levels are associated with the extent of functional connectivity within and between Resting State Networks, specifically, the anterior attentional neural centers partaking in *task positive* brain network, such as DLPFC and ACC, and the central-posterior structures supporting the *default mode network* (100). Sleep deprivation specifically has been shown to impair top-down mechanisms of control, providing support for the findings presenting here, specifically within the top-down control that governs task execution and cognitive control (101).

#### 4.2.4. Social jetlag and chronobiological influences

The results of the measure of social jetlag (SJL), which is used to measure the difference between social and biological time, reported that on average, the weekly difference between mid-sleep on school days and weekends was approximately 123 min (18). Social jetlag of each day of the week was determined with the same calculation as the weekly social jetlag, however the single day was used instead of the average of the week. In contrast to other findings on weekly SJL in the literature, we found that daily SJL correlated only modestly with processing speed but was very much consistent with the pattern in accuracy.

Largely, the SJL measure in nature considers the social and biological factors influencing sleep. The measure is often cited in work that may directly or indirectly posit a bottom-up approach to factors influencing sleep patterns and cognitive performance, with emphasis on subcortical structures such as the Suprachiasmatic nucleus (SCN). We agree with this position, however we offer an addition to this approach which also takes into account the top-down influence of cortical neural networks, which likely also contributes to the overall picture of sleep related behavior (101). Previous research has shown that naturally, sleep deprivation or debt can lead to cognitive decline via top-down attention control deficits (102), while it has also been shown that sleep deprivation may not elicit deficiencies in bottom-up cognitive control (101). An apt metaphor is describing the contributions of both bottom-up and top-down influence over cognitive function as a *balancing act*. This posits the idea that while SJL is a valuable measure to determine the discrepancy that arises from social vs. biological time differences, in this study it is not necessarily effective in predicting cognitive correlates of sleep deprivation, since they may not arise from a strictly biological/bottom-up mechanism. This latter interpretation of our findings is supported by evidence suggesting that the extent to which sleep deprivation affects performance may be attenuated by individual differences in cognitive control driven by top-down (PFC) attentional and executive processes (103). Namely, individuals and students who have stronger executive attention functions may exert their cognitive control and show stable, optimal performance even after sleep deprivation, but those with weak cognitive control may be influenced severely by sleep deprivation, namely, show much lower performance (104–106). This relationship has been exemplified in findings showing that the extent of vulnerability to sleep deprivation is correlated with the severity of some cognitive control outcomes associated with ADHD (107, 108).

#### 4.2.5. Conclusions and limitations

Taken together, the results offer converging evidence that the participants may have not been able to adjust to the early sleep pattern on Monday. By Monday afternoon, participants may have been sleepier than on Wednesday afternoon, which could be explained by the challenge in attenuating the low-frequency EEG activity in congruent trials. Therefore, the present findings suggest that delayed sleep patterns in combination with conflicting circadian preference (chronotype) may account for the adolescents’ differences in ERPs and cognitive performance, thereby partly and possibly linking sleep patterns related to SSTs to specific alteration of adolescent circadian EEG activity. Furthermore, SJL was only partially associated with the pattern of findings, we put forth the additional suggestion that there is a shared contribution of circadian cycle and sleep pattern to cognitive control, such that in this instance, there seems to be an equally important contribution from sleep pattern associated with homeostatic regulation learned habits as from circadian cycle.

A limitation of the present study is that we interpreted the diary question of turning lights off as a self-reported sleep onset time when this is closer to a “bedtime” measure than a “sleep-onset” measure. In addition, bedtime recall is difficult from diary. We did not have other measures of sleep (actigraphy, parental report) to corroborate the bedtime estimation. The issue is that the nature of the circadian delay observed in the sample and the assumed circadian preferences depended on few bedtime measures. Yet, we note that currently there is no better measure of sleep onset than converging lines of evidence from subjective self-reports and objective indices (such as polysomnography), any standalone self-report or observational measure conditional on wakefulness [including actigraphy, see for example (109, 110)] implies some reliability limitations, as it yields only a certain degree of precision. Nevertheless, there is a consensus that diary measures have some validity in capturing basic underlying properties which are sought to be measured, especially in ecological conditions, which fits the purposes of this study. Consequently, the validity and reliability of our findings largely rests on and should be assessed in the light of the converging evidence of *relationships* within consistent but different diary data collected at different moments, and between the latter sources and the converging objective EEG/ERP findings.

Overall, one plausible explanation for our findings is that the reduced accuracy and dampened ERP response observed on Monday morning may be due to combined influences of delayed sleep pattern and decoupled chronotype influenced by SSTs (14, 58, 63). Slowed ERPs in the afternoon of midweek, despite improved performance, could be due to fatigue associated with sleep deprivation. The results suggest that students are alert enough to perform cognitive tasks with relative accuracy albeit with reduced neural activity that continues throughout the day. Therefore, to compensate the effects of SSTs, adolescents may have to engage in more effortful cognitive processing in the early-week mornings and be more prone to fatigue by mid-week afternoons. Alternatively, another explanation for the observed ERP effects is the combination contribution of sleep misalignment and early SST on the Sunday–Monday transition, and the growing sleep deprivation over the course of the week. In this case, both top-down cortical influences of behavior that result from sleep deficiency, and bottom-up influences that arise from social/biological disruption may contribute to the overall profile of the ERPs. We posit that this interpretation may be perhaps more justifiable from a chronobiological perspective, however measures such as SJL did not sufficiently support a social/biological misalignment as being the sole account for the observed pattern of findings.

The present findings have implications for the influences of SSTs on adolescents’ brain activity and neurocognitive functioning, adding to the growing evidence that SSTs, however defined as “early,” may have serious impacts on adolescent student wellbeing. Despite the study limitations, these results are among only a handful of studies which have used objective measures to study the complex networks of relationships influencing ecological adolescents’ sleep patterns and cognitive functioning during school hours. With further refinements, the methodological and measurement approaches used in this study could offer additional tools to measure and monitor the influences of SSTs, possibly leading to robust findings that could help inform policy changes and/or possibly the alternative of ameliorative strategic interventions.

The present findings have implications for education. A school’s ultimate decision on testing times, exam schedules, and SSTs can be informed using evidence of how cognitive ability and control may vary throughout the day and the week. In one instance, the misalignment between adolescent chronotype and SST may be corrected by pushing back SSTs to prevent the development of a large SJL on the weekend. In so doing, the cognitive deficits which arise because of SJL, or the so-called ‘Monday Effect’, can be reduced or potentially erased. Indeed it is already documented that pushing back SSTs improves academic performance (111). By understanding the factors that have been shown to alter cognitive performance throughout the day and week, there can also be methodological approaches to planning exams and course times as well as the extent of cognitive vulnerability to sleep deprivation. Based on the data we have presented here, students attending exams and classes on Monday morning may underperform as compared to attending them on Wednesday afternoon. Thus, a student who was assigned exams that were all within the first few hours of the school day might experience more cognitive deficit than the same student assigned exams later in the week and in the afternoon. Naturally, these findings are constrained by the specific context. The present study included only female participants and thus expanding its findings to a more general population that is not restricted to females may find generalizability not confronted by developmental differences across the sexes. It is important to note, however, that it should be possible to apply some of our findings even to male youth, since it could be predicted that the effects highlighted in our data may apply to males later in their development, so they could be predicted as occurring in a similar fashion for males albeit delayed a few years later.

Beyond development, natural behavioral tendencies arise that may also reduce generalizability of these findings (e.g., caffeine intake, sleep schedule) (112). Despite this, however, consensus of SSTs and testing/learning schedules may benefit from taking into consideration the effect on cognition that sleep, and its week/weekend mismatch, may produce. In a wider context, cultural differences must also be taken into consideration when interpreting these results. In a paper investigating cross-cultural sleep duration differences between Japanese and Canadian students, it was found that Japanese participants reported better health and were less tired, despite having less sleep than European Canadians (113). This finding appears paradoxical considering that populations living in Western cultures sleep longer on average, due to the perspective that sleep is important for health. It does, however, fit into the general findings that East Asian populations sleep less than Western populations, possibly due to cultural opinions on sleep and its importance. This mismatch, and the general inconsistency in sleep duration across the world, means that findings for a Canadian adolescent population are relevant for this sample, but not necessarily another. Even within Canada, though, there are factors which must be taken into consideration. One particularly blatant influence for a northern country is that the amount of sunlight (as a function of sunrise and sunset) varies considerably from the equator to the poles (114). We recognize that these numerous factors limit our research’s generalizability to cultures, countries, and even subpopulations of students living in other Canadian regions.

## Data availability statement

The raw data supporting the conclusions of this article will be made available by the authors, without undue reservation.

## Ethics statement

The studies involving human participants were reviewed and approved by Carleton University Research Ethics Institutional Board. Written informed consent to participate in this study was provided by the participants’ legal guardian/next of kin.

## Author contributions

AD’A, GGa, and W-HY contributed to conception and design of the study. GGa collected data. PD, TD, W-HY, and GB organized the database. PD, TD, and GB curated visualization. AD’A, W-HY, and GB performed the statistical analysis. AD’A wrote the first draft of the manuscript. AD’A, GB, W-HY, GGo, and GL, re-wrote and revised sections of the manuscript. GB formatted all materials. All authors contributed to the manuscript numerous further rounds of revisions, read, and approved the submitted version.

## Funding

This research was funded by a Canada Research Chair award as well as seed funding from the Human Early Learning Partnership, University of British Columbia.

## Conflict of interest

The authors declare that the research was conducted in the absence of any commercial or financial relationships that could be construed as a potential conflict of interest.

## Publisher’s note

All claims expressed in this article are solely those of the authors and do not necessarily represent those of their affiliated organizations, or those of the publisher, the editors and the reviewers. Any product that may be evaluated in this article, or claim that may be made by its manufacturer, is not guaranteed or endorsed by the publisher.

## Footnotes

^1^ http://www.pre.ethics.gc.ca/pdf/eng/tcps2-2014/TCPS_2_FINAL_Web.pdf

^2^ https://kamloops.weatherstats.ca/charts/sun-monthly.html
