# Supplementary material for: Cognitive control, bedtime patterns, and testing time in female adolescent students: behavioral and neuro-electrophysiological correlates
Source: Front Public Health. 2023 Jun 19;11:1022731. doi: 10.3389/fpubh.2023.1022731 (PMC10315662; doi:10.3389/fpubh.2023.1022731)
Supplement: Supplementary file 1 [file Table_1.DOCX]

Supplementary Material

# Supplementary Data

NA

# Supplementary Figures and Tables

## Supplementary Figures


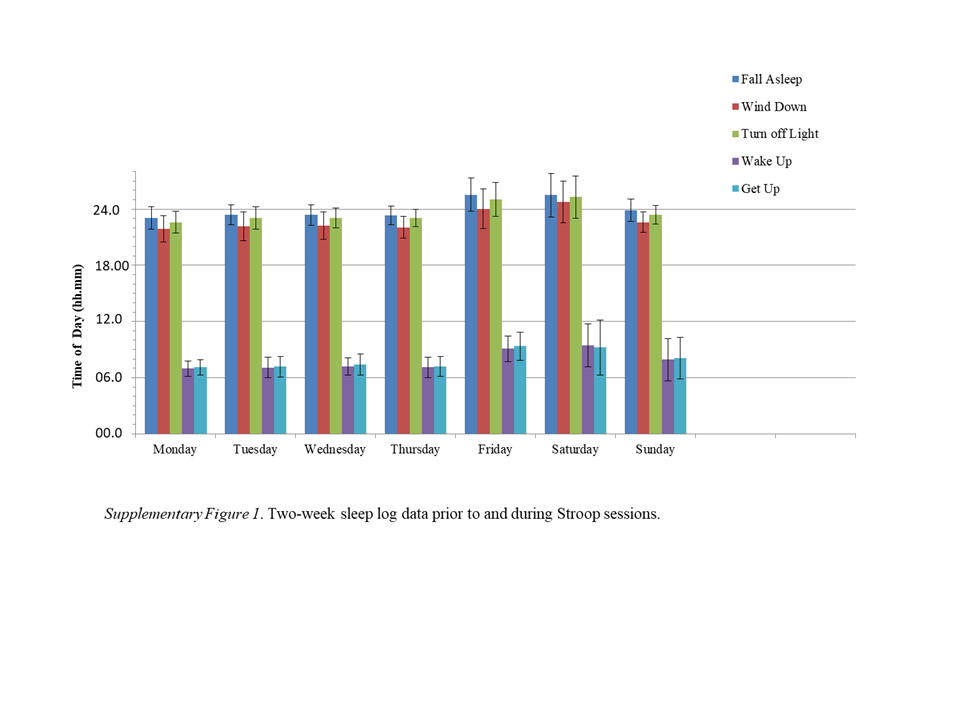
**Supplementary Figure 1.** Two-week sleep log data prior to and during Stroop sessions showing time of day for each of falling asleep, winding down, turning of the lights, waking up, and getting up. Data is presented for each day of the week. Error bar represents standard error of the mean.

## Supplementary Tables

## S.Table 1. Study design, samples and schedule of testing.

|  | Testing day | | | |
| --- | --- | --- | --- | --- |
|  | (Week k) | | (Week k + 1) | |
|  | Monday | Wednesday | Monday | Wednesday |
| Testing time |  |  |  |  |
| Morning | Subgroup 1  (Grade 11) | Subgroup 2  (Grade 12) | Sub-Group 2  (Grade 12) | Sub-Group 1  (Grade 11) |
| Afternoon | Subgroup 3  (Grade 12) | Sub-Group 4  (Grade 11) | Sub-Group 4  (Grade 11) | Sub-Group 3  (Grade 12) |

Note. Within each cell, n = 3. Within the same grade subgroups, participants were matched for age (between-subjects difference criterion ≤ ± 4 months).

**S.Table 2.** Mean ratings (and standard deviations) of perceived quality of sleep and alertness as a function of week progression.

|  | Monday | Tuesday | Wednesday | Thursday | Friday | Saturday | Sunday |
| --- | --- | --- | --- | --- | --- | --- | --- |
| Sleep  Quality^a^ | 3.58  (0.94) | 3.75  (0.98) | 3.61  (0.68) | 3.50  (0.96) | 3.66  (1.25) | 3.64  (0.83) | 3.86  (0.81) |
|  |  |  |  |  |  |  |  |
| Alertness^b^ | 3.11  (1.05) | 3.55  (0.84) | 3.32  (1.19) | 2.93  (1.30) | 3.41  (1.33) | 3.75  (1.07) | 3.38  (1.06) |

Note. Values in the table represent ratings on a 5-point Likert scale.^a^ n = 18; ^b^ n = 19.
